# MicroRNA Expression Analysis of In Vitro Dedifferentiated Human Pancreatic Islet Cells Reveals the Activation of the Pluripotency-Related MicroRNA Cluster miR-302s

**DOI:** 10.3390/ijms19041170

**Published:** 2018-04-12

**Authors:** Guido Sebastiani, Giuseppina Emanuela Grieco, Noemi Brusco, Giuliana Ventriglia, Caterina Formichi, Lorella Marselli, Piero Marchetti, Francesco Dotta

**Affiliations:** 1 Diabetes Unit, Department of Medicine, Surgery and Neurosciences, University of Siena, Fondazione Umberto Di Mario ONLUS c/o Toscana Life Science, 53100 Siena, Italy; sebastianiguido@gmail.com (G.S.); giusy.grieco.90@gmail.com (G.E.G.); noemibrusco91@gmail.com (N.B.); giulianaventriglia@gmail.com (G.V.); catefo@libero.it (C.F.); 2 Department of Clinical and Experimental Medicine, University of Pisa, 56126 Pisa, Italy; lorella.marselli@med.unipi.it (L.M.); piero.marchetti@med.unipi.it (P.M.)

**Keywords:** human pancreatic islets, dedifferentiation, microRNA, miR-302

## Abstract

β-cell dedifferentiation has been recently suggested as an additional mechanism contributing to type-1 and to type-2 diabetes pathogenesis. Moreover, several studies demonstrated that in vitro culture of native human pancreatic islets derived from non-diabetic donors resulted in the generation of an undifferentiated cell population. Additional evidence from in vitro human β-cell lineage tracing experiments, demonstrated that dedifferentiated cells derive from β-cells, thus representing a potential in vitro model of β-cell dedifferentiation. Here, we report the microRNA expression profiles analysis of in vitro dedifferentiated islet cells in comparison to mature human native pancreatic islets. We identified 13 microRNAs upregulated and 110 downregulated in islet cells upon in vitro dedifferentiation. Interestingly, among upregulated microRNAs, we observed the activation of microRNA miR-302s cluster, previously defined as pluripotency-associated. Bioinformatic analysis indicated that miR-302s are predicted to target several genes involved in the control of β-cell/epithelial phenotype maintenance; accordingly, such genes were downregulated upon human islet in vitro dedifferentiation. Moreover, we uncovered that cell–cell contacts are needed to maintain low/null expression levels of miR-302. In conclusion, we showed that miR-302 microRNA cluster genes are involved in in vitro dedifferentiation of human pancreatic islet cells and inhibits the expression of multiple genes involved in the maintenance of β-cell mature phenotype.

## 1. Introduction

It has been previously demonstrated that in vitro culture of native adult human pancreatic islets derived from non-diabetic donors resulted into the generation/expansion of an undifferentiated cell population [1]. Several studies demonstrated that the resulting cell population derives from an epithelial to mesenchymal transition (EMT) program which induces specialized islet cells to lose endocrine pancreatic markers (dedifferentiation) while acquiring a mesenchymal/multipotent phenotype [2,3,4]. Moreover, additional evidence from in vitro human β-cell lineage tracing experiments demonstrated that dedifferentiated cells derive also from β-cells, thus representing a potentially ideal in vitro model of β-cell dedifferentiation [5,6,7].

The dedifferentiation process plays an important role during embryonic development, generating cells with stem-cell properties. Indeed, most adult tissues arise from a series of conversions of epithelial cells into dedifferentiated cells and from the reverse process [8]. However, whether dedifferentiation represents a physiological or a pathological process contributing or not to islet neogenesis/regeneration or dysfunction remains to be fully established [9,10]. Recently, several studies demonstrated that a phenomenon resembling β-cell dedifferentiation occurs both in type 1 (T1D) and type 2 diabetes (T2D) [11,12,13,14,15,16]. Indeed, several endocrine-positive/hormone-negative islet cells have been identified by analyzing pancreas sections from patients with T2D [11]. However, even though dedifferentiated cells have been detected using multiple histological approaches, their molecular architecture has not yet been fully elucidated. Several efforts pursuing this goal have been made by analyzing a number of pathways underlying the phenotype loss in in vitro dedifferentiated β-cells.

MicroRNAs are short endogenous RNA, 19–24 nucleotides long, which negatively regulate gene expression through their binding to the 3′UTR of mRNAs target with subsequent mRNA degradation or translational inhibition [17]. Depending on their target genes, expression levels, and cell/tissue distribution, microRNAs may regulate both stem-cell phenotype induction and differentiated/specialized cell phenotype functional maintenance [18]. Deregulation/modulation of the expression of specific microRNAs may alter or totally change the cell fate, thus making them optimal potential targets to modulate cells phenotype plasticity [19,20]. Specifically, several microRNAs have been identified as peculiar modulators of β-cell phenotype and function [21,22]; indeed, it has been previously demonstrated that several microRNAs, including miR-375 as well as microRNAs defining β-cell/epithelial cell phenotype (e.g., miR-200s, miR-30s), were downregulated during in vitro dedifferentiation, underlining their pivotal role in the β-cell phenotype maintenance [23,24]. Therefore, it is conceivable to hypothesize that during this process several other microRNA families may play a role in the induction of a dedifferentiated phenotype.

Here, we identified the activation of the pluripotent-specific miR-302s microRNA cluster during in vitro dedifferentiation of non-diabetic human pancreatic islet cells leading to the hypothesis of a potential role for such microRNAs in the regulation and induction of β-cell dedifferentiated phenotype.

## 2. Results

### 2.1. MicroRNA Expression Profiles of In Vitro Dedifferentiated Islet Cells

We and others have previously demonstrated that prolonged human pancreatic islets culture results in delamination, adhesion, and migration of endocrine cells from islet native architecture; such morphological changes are associated to the loss of pancreatic-endocrine phenotype, in a process resembling epithelial–mesenchymal transition (EMT) [1,5]. Although the peculiar mechanisms and transcriptome analysis of this process have been clearly reported, the contribution of microRNA is poorly understood and deserves further analysis.

To this aim, we dedifferentiated in vitro human pancreatic islets cells (see Methods section and reference [1]) derived from *n* = 3 non-diabetic organ donors (Age 63.3 ± 23.3 year; BMI 24.8 ± 1.3 Kg/m^2^) and compared them to fully differentiated human native islet cells (*n* = 3) (Age 54.6 ± 21.3 year; BMI 25.4 ± 1.8 Kg/m^2^) (extended donors characteristics reported in Supplementary Table S1).

**Figure 1:**
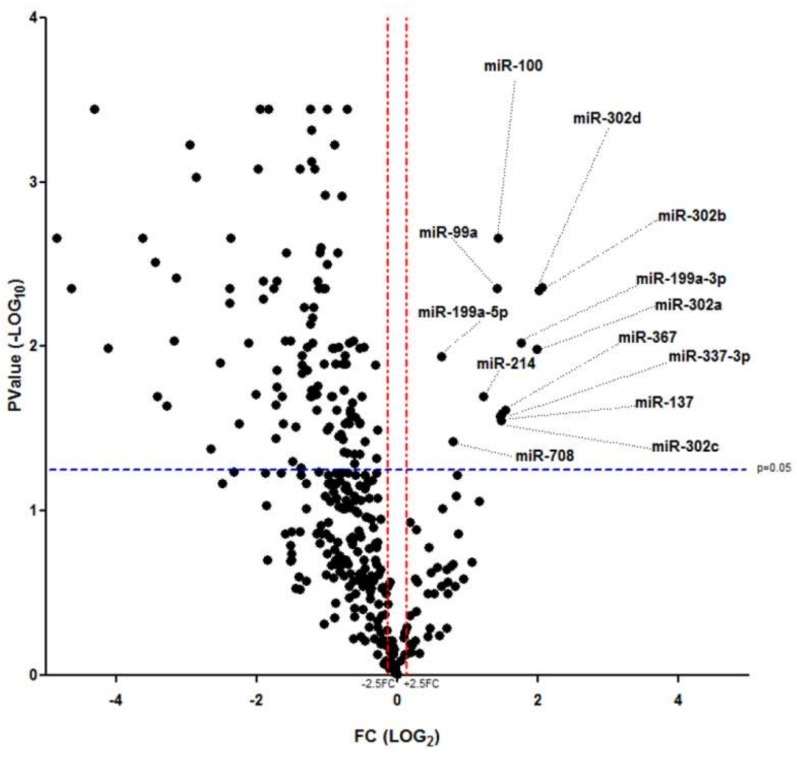
MicroRNA profiling of dedifferentiated human islet cells. Volcano plot analysis showing differentially expressed microRNAs in in vitro dedifferentiated human pancreatic islet cells vs. human native pancreatic islets. Black dots represent detected microRNAs based on the relative mean fold change (-Log FC) and *p*-values. Fold change cutoff (red lines) was set at 2.5-fold while *p*-values cutoff (blue line) was set at 0.05 based on FDR corrected Student’s *t*-test on normally distributed dCt values. The position and the identification name of upregulated microRNAs on the volcano plot is indicated by black dotted lines.

Firstly, in order to confirm the loss of differentiated/mature endocrine phenotype and to set the stage for global microRNA analysis, we evaluated the expression of marker genes associated to endocrine-pancreatic and to undifferentiated/mesenchymal phenotype, both in human native pancreatic islets and in dedifferentiated islet cells. As expected, the results showed a significant reduction of endocrine pancreatic marker genes expression (INS, GCG, SST, NEUROD1, PDX1) and a concomitant activation of undifferentiated/mesenchymal phenotype associated markers (NES, VIM, ZEB1, ZEB2, TWIST1) (Supplementary Figure S1a,b). Subsequently, we analyzed the expression profile of microRNAs (768 microRNAs) in human pancreatic islets derived from *n* = 3 non-diabetic multiorgan donors and in *n* = 3 in vitro expanded and dedifferentiated islet-derived cells. A total of 342 microRNAs were detected (cutoff Ct < 35.0 in all replicates of at least one group) (Supplementary Figure S2) and 123 of them resulted differentially expressed (fold change cutoff <0.35, >2.5, *p* < 0.05 unpaired *t*-test False Discovery Rate (FDR) corrected); of these, 110/123 (89.4%) were significantly downregulated (for the complete list of downregulated microRNAs see Supplementary Figure S3) and 13/123 (10.5%) were upregulated in dedifferentiated islet-derived cells vs. fully differentiated/mature islet cells (Figure 1).

In accordance with previous reports, among downregulated microRNAs in dedifferentiated islet-derived cells, we detected miR-375 and miR-141-200 families, previously associated with β-cell function and epithelial phenotype, which clustered together in the global hierarchical clustering analysis (see detail in Supplementary Figure S4a). Furthermore, within this group of microRNAs we also detected islet-enriched/β-cell specific microRNAs and islet/β-cell development associated microRNAs (miR-9, miR-155, miR-30a, miR-30d, miR-25) (Supplementary Figure S3), thus additionally confirming the loss of β-cell/endocrine-pancreatic phenotype upon in vitro dedifferentiation. Despite the quite large amount of downregulated microRNAs, those found upregulated represented the minor part (13 microRNAs: miR-99a, miR-100, miR-137, miR-199a-5p, miR-199a-3p, miR-214, miR-302a-3p, miR-302b-3p, miR-302c-3p, miR-302d-3p, miR-367, miR-337-3p, and miR-708) (detailed hierarchical clustering analysis in Supplementary Figure S4b).

Collectively, these results demonstrate that 35.8% of detected microRNAs were differentially expressed upon in vitro dedifferentiation of non-diabetic human pancreatic islets and that only a minor part of those differentially expressed (13/123 microRNAs) were upregulated during this process, thus possibly representing biomarkers of dedifferentiation. We focused on this last set of microRNAs, since their upregulation during dedifferentiation may target key genes involved in islet/β-cell function and phenotype maintenance, thus leading to their downregulation and to the potential loss of a differentiated/mature endocrine phenotype.

### 2.2. miR-302 MicroRNAs Expression Is Switched-On during Islet Cells Dedifferentiation

**Figure 2:**
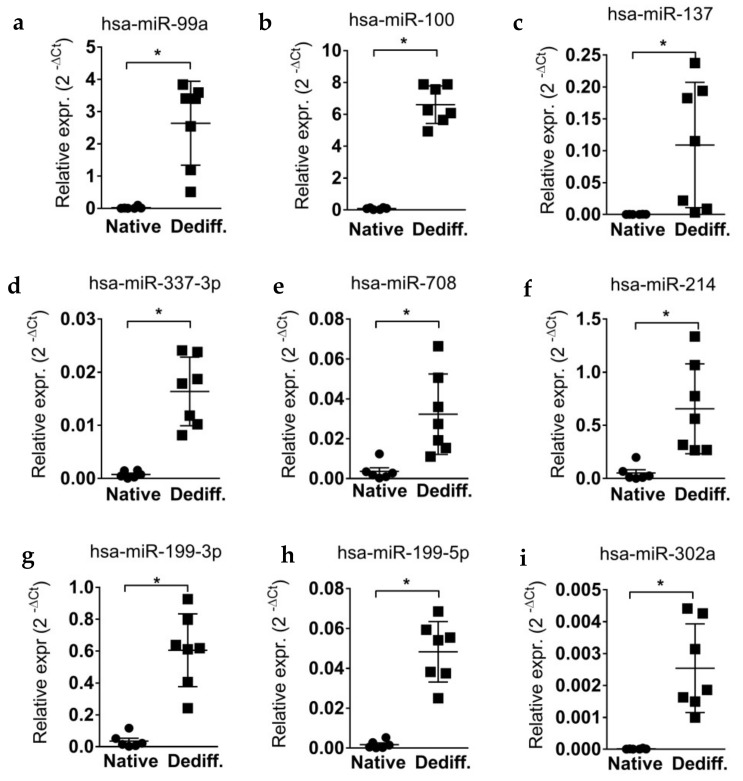

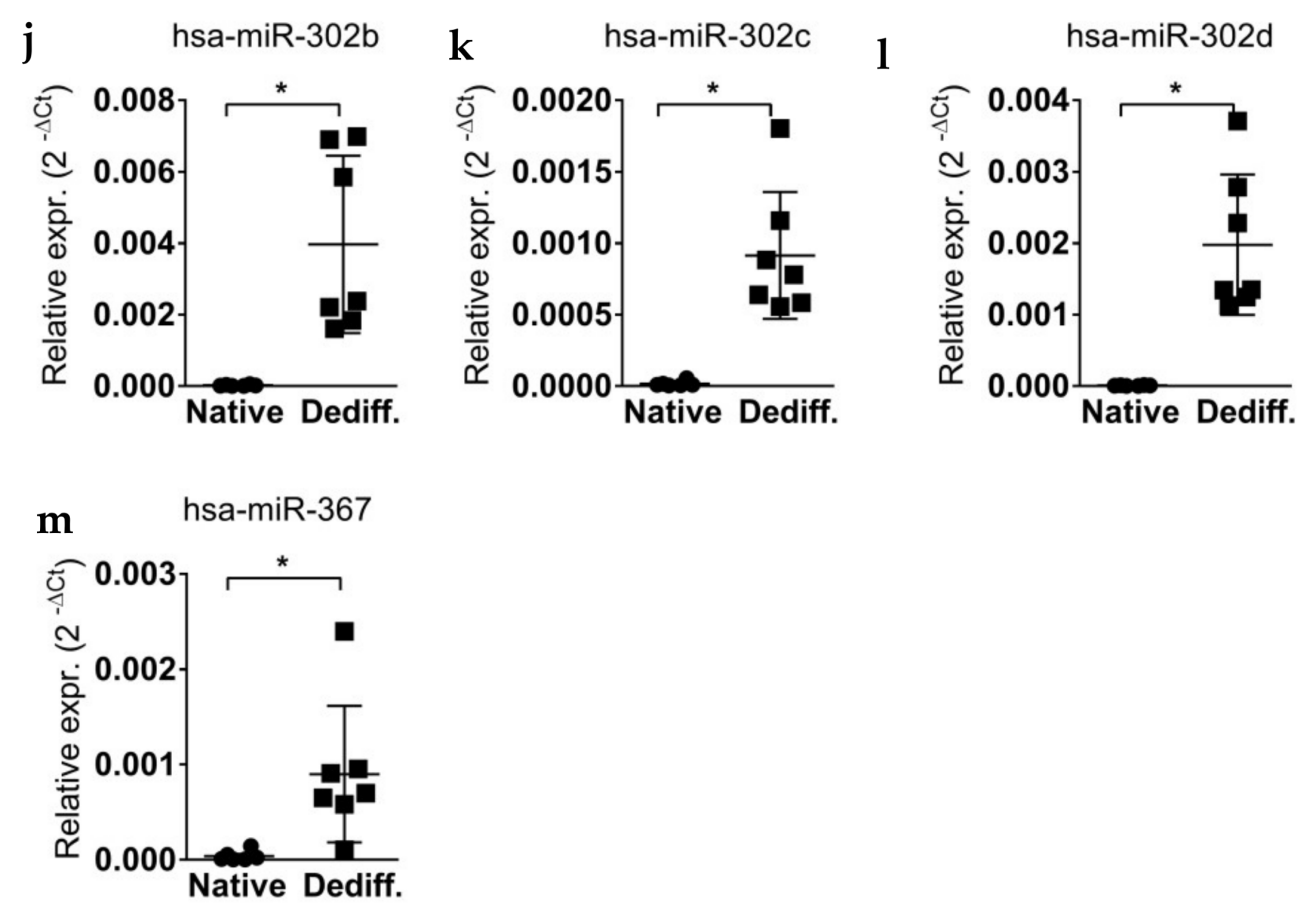
Validation of differentially expressed microRNAs in dedifferentiated islet cells. Stem–loop RT-qPCR single assay validation of 13 identified upregulated microRNAs in dedifferentiated human pancreatic islet cells. Single assay RT-qPCR validation of *n* = 6 native human islets and *n* = 7 islet-derived mesenchymal cells of miR-99a (**a**), miR-100 (**b**), miR-137 (**c**), miR-337-3p (**d**), miR-708 (**e**), miR-214 (**f**), miR-199-3p (**g**), miR-199-5p (**h**), miR-302a (**i**), miR-302b (**j**), miR-302c (**k**), miR-302d (**l**), and miR-367 (**m**)*.* Data are reported as normalized 2^−Δ*C*t^ values together with mean ± SD. Mann–Whitney U test, * *p* < 0.05.

In order to confirm the upregulation of the 13 differentially expressed microRNAs in dedifferentiated islet cells, we validated their expression using single assay stem–loop RT-qPCR in the same samples previously profiled and in additional human pancreatic native islet preparations and dedifferentiated islet-derived cell samples (a total of *n* = 6 native human pancreatic islet samples; *n* = 7 dedifferentiated islet-derived cell samples) (donors characteristics reported in Supplementary Table S1). The analysis confirmed the results obtained in the profiling stage (Figure 2), thus revealing the significant upregulation (*p* < 0.05, non-parametric Mann–Whitney U test) of those microRNAs upon in vitro dedifferentiation of non-diabetic human pancreatic islet cells.

Of note, among upregulated microRNAs we identified five microRNAs belonging to miR-302s cluster [25], whose expression was low/null in native/mature islets but strongly and significantly induced upon dedifferentiation (Figure 2i–m). miR-302s have been described to be highly involved in pluripotent-stem cell maintenance and in the acquisition of undifferentiated phenotype [26,27], thus potentially suggesting an unprecedented role for these microRNAs in islets/β-cells dedifferentiation and reinforcing the view of microRNAs as active participants in the loss of islets/β-cells phenotype.

### 2.3. Upregulated MicroRNA Target Key Genes with Multiple Roles in Endocrine/Epithelial Phenotype Maintenance

**Figure 3:**
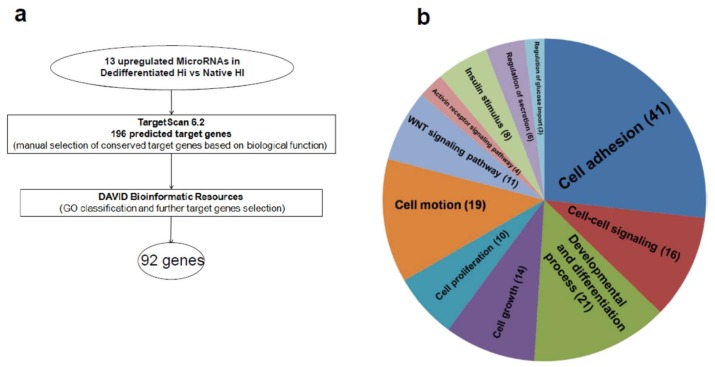
(**a**) Scheme diagram of bioinformatic analysis workflow using Targetscan 6.2 and David 6.7 algorithms. (**b**) Graphical representation of GO terms classification of predicted target genes of upregulated microRNAs: GO Term ID alongside with number of gene included are reported.

In order to identify the pattern of target genes regulated by the entire set of upregulated microRNAs in dedifferentiated islet-derived cells and potentially involved in this process, we adopted a bioinformatic approach using a microRNA-target gene prediction algorithm (Targetscan 6.2) followed by a gene ontology (GO) classification profiling (David 6.7) (bioinformatic workflow scheme in Figure 3a). Overall, for the 13 upregulated microRNAs, we identified 196 target genes involved in differentiation, cell-adhesion or proliferation functions. In order to obtain a more in depth functional classification, the set of identified predicted target genes were analyzed using David 6.7 (Figure 3a).

The results showed that most of them belong to “cell-adhesion” (GO0007155, *p* = 5.18 × 10^−26^), “cell–cell signaling” (GO0007267, *p* = 2.88 × 10^−5^) and “positive regulation of development” (GO0051094, *p* = 9.12 × 10^−15^) (Figure 3b and Table 1).

Additionally, we further selected some of them based on their functional relevance and on David 6.7 classification; such final evaluation resulted in a stringent selection of 92 predicted target genes strongly associated with the main biological processes involved in dedifferentiation/loss of phenotype.

**Figure 4:**
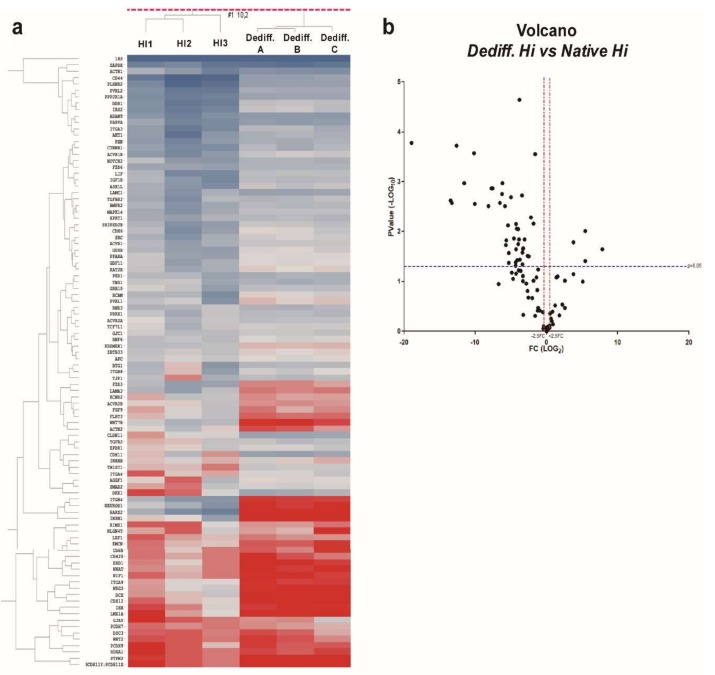
MicroRNA target genes expression profiling. (**a**) Hierarchical clustering analysis showing expression levels of selected target genes in human native islets (Hi1, Hi2, Hi3) and dedifferentiated islet cells (Dediff.A, Dediff.B, Dediff.C). Expression values of each target gene are reported as ΔCt and fitted into color scale (blue: high expression; red low/null expression). (**b**) Volcano plot analysis showing differentially expressed genes (selected based on the functional bioinformatic analysis) in dedifferentiated human islet cells (*n* = 3) vs. native islets (*n* = 3). Single black dots represent each detected gene based on the relative mean fold change (-Log FC) and *p*-values. Fold change cutoff (red lines) was set at 2.5-fold while *p*-values cutoff (blue line) was set at 0.05 based on Student’s *t*-test on normally distributed ΔCt values.

To determine whether the 92 selected target genes were differentially expressed during in vitro dedifferentiation, we evaluated their levels in human native islets (*n* = 3) and dedifferentiated islet cell samples (*n* = 3), initially analyzed for microRNA expression profiles. The results revealed that 48/92 genes were significantly differentially expressed. Among them, 44/92 genes were downregulated while 4/92 genes were upregulated in dedifferentiated islet cells vs. human native pancreatic islets (Figure 4a,b).

Collectively, these results demonstrate that upregulated microRNAs upon in vitro dedifferentiation of human pancreatic islets may control several genes involved in epithelial cell adhesion, cell–cell signaling, and β-cell phenotype maintenance; such genes were shown to be mostly downregulated during this process and their differential expression is in line with the upregulation of the targeting microRNAs.

### 2.4. miR-302 MicroRNA Target Several Genes Involved in β-Cell Function and Are Regulated by Cell–Cell Contacts during In Vitro Dedifferentiation of Human Islets

**Figure 5:**
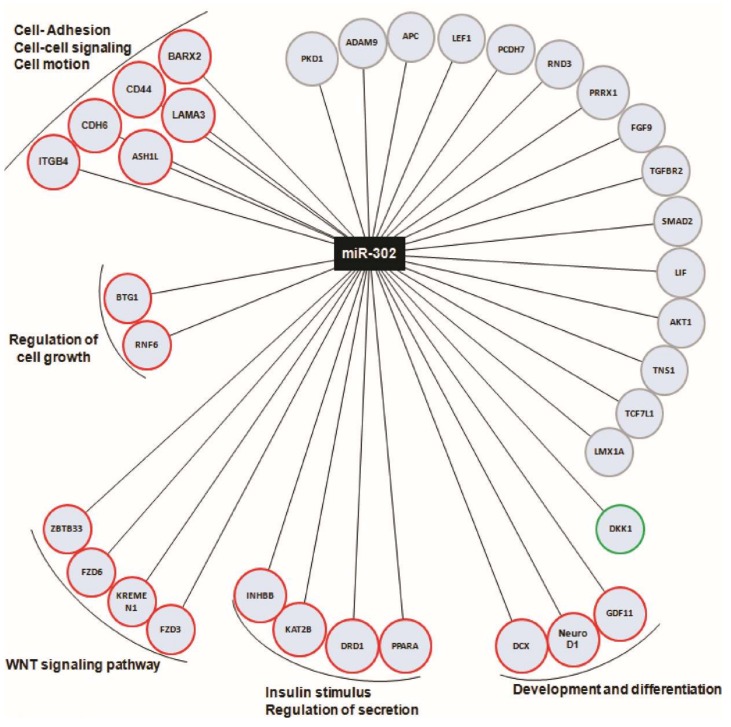
miR-302s target genes are involved in the loss of epithelial phenotype. miR-302 family are downregulated in islet-derived dedifferentiated cells respect to native human islets and are mainly involved in cell adhesion and motion, cell–cell signaling, cell growth, development and differentiation, WNT signaling pathway and insulin stimulus, as well as regulation of secretion. The scheme reports the entire set of analyzed miR-302s target genes; downregulated genes in islet-derived dedifferentiated cells are reported in red, while those upregulated are in green. Grey highlighted miR-302s target gene did not show any significant differential expression upon dedifferentiation.

We observed that among 92 analyzed genes in human pancreatic islets vs. in vitro dedifferentiated islet cells, 34 are putatively targeted by miR-302s microRNAs. Among those, 19/34 were downregulated and in line with the upregulation of miR-302s, 14/34 did not show any significant differential expression and 1/34 resulted upregulated upon in vitro dedifferentiation (Figure 5).

We further categorized downregulated miR-302 target genes and demonstrated that most of them are involved in cell–cell adhesion (ITGB4, CDH6, CD44, BARX2, ASH1L, LAMA3), in β-cell phenotype control and/or function (NeuroD1, KAT2B, PPARα, DRD1) or in the control of WNT/β-catenin signaling (FZD3, FZD6, ZBTB33, KREMEN1), thus suggesting that the transcriptional activation of miR-302s is associated with the loss of phenotype of human pancreatic islet cells.

**Figure 6:**
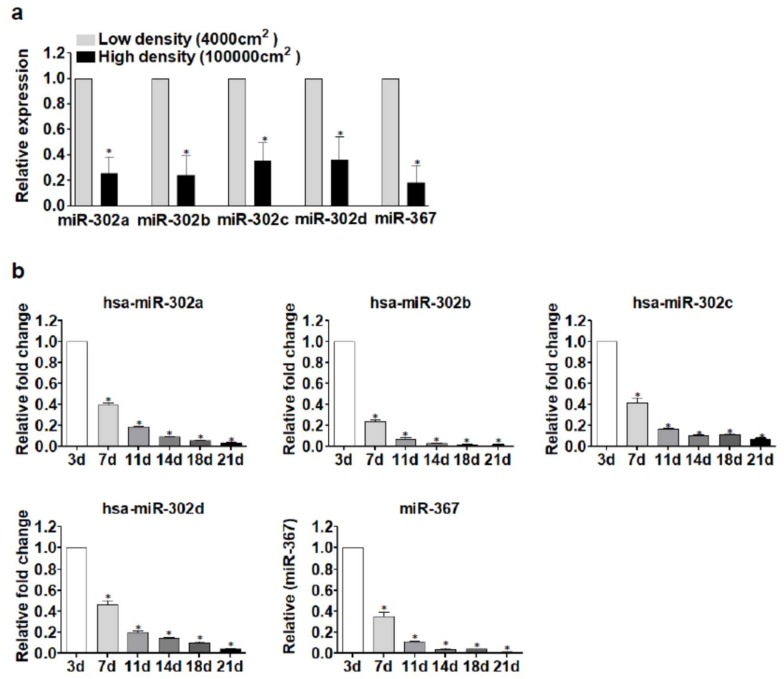
Cell–cell contacts contribute to the modulation of miR-302s expression. (**a**) miR-302 family members expression evaluation using stem–loop RT-Real Time PCR in dedifferentiated islet cells cultured at different density (4000 cells/cm^2^ vs. 100,000 cells/cm^2^) for 48 h. Values are reported as mean fold change ± SD vs. low density plated dedifferentiated islet cells. *n* = 3 independent experiments using three different human islets-derived dedifferentiated cells preparations, *p*-value Student’s *t*-test * *p* < 0.05. (**b**) Evaluation of miR-302s expression during re-differentiation of human islet-derived dedifferentiated cells, starting from day 3 and evaluated at day 7, 11, 14, 18, and 21. Values are reported as mean fold change ± SD vs. day 3 of re-differentiation. *n* = 3 independent experiments using three different human islets-derived dedifferentiated cells preparations, *p*-value Student’s *t*-test * *p* < 0.05.

Previously, miR-302 microRNAs have been demonstrated to be highly expressed in pluripotent stem cells [26,27] where they are transcriptionally controlled by Nanog and Oct3/4 transcription factors [28,29,30]. We hypothesized that the potential activation of such stem cell factors during human islet in vitro dedifferentiation may drive the activation of miR-302s. However, the expression analysis of Nanog and Oct3/4 in in vitro dedifferentiated islet cells vs. human native islets showed low/null expression of these factors without any significant transcriptional activation during dedifferentiation. More recently, the transcription of miR-302 microRNA cluster genes have also been demonstrated to be under the control of Wnt/β-catenin signaling; additionally, other studies demonstrated the activation of this signaling pathway during in vitro human islet cells dedifferentiation [31,32,33]. Since the modulation of Wnt signaling pathway is promoted also by sequestration/release of β-catenin from cell–cell adhesion proteins (e.g., Cadherins) [34], we hypothesized that establishment or disruption of cell–cell contacts may modulate the expression of miR-302s. Therefore, to verify such hypothesis, we cultured dedifferentiated islet cells at low (4000 cells/cm^2^) or high cell density (100,000 cells/cm^2^) in order to establish whether forced cell–cell contacts could induce a reduction of miR-302s expression. Indeed, the expression of miR-302s was significantly lower in high cell density vs. low density culture (Figure 6a), suggesting that cell–cell contacts can modulate miR-302s expression through Wnt or other signaling pathways.

We and others have previously demonstrated that in vitro expanded and dedifferentiated islet cells are able to re-differentiate upon a specific 21 days culture protocol [1,35]; such differentiation program toward endocrine-pancreatic phenotype is also characterized by re-establishment of cell–cell contacts with the formation of pseudo-islets, starting from day 3 of differentiation at which most dedifferentiated cells maintained cell–cell contact [1]. Therefore, to determine whether cell–cell contacts may re-establish miR-302s expression, we analyzed their expression during re-differentiation phase at different time points (-3, -7, -11, -14, -18, -21 days of differentiation). Indeed, expression of miR-302s during re-differentiation was shown to be progressively reduced alongside with the ongoing re-establishment of cell–cell contacts (Figure 6b).

Collectively, these results underline the potential role of pluripotent-associated microRNA, miR-302, during in vitro dedifferentiation of human pancreatic islets and suggest that cell–cell contacts may contribute to the activation of their expression.

## 3. Discussion

Human β-cell dedifferentiation, or loss of phenotype, has been recently addressed as a new potential mechanism of β-cell dysfunction in diabetes pathogenesis [36]. Importantly, it has been suggested that this process can be reversible, thus opening the possibility of a re-establishment of a fully functional β-cell phenotype as a therapeutic approach. However, the obvious difficulties of molecular analysis of dedifferentiated cells, mainly due to the lack of evident biomarkers of this process, is slowing down the discovery of the detailed mechanisms associated to the loss of β-cell phenotype. Therefore, in vitro modeling of dedifferentiation could represent an ideal opportunity to uncover potential in situ biomarkers able to identify dedifferentiated cells.

Human pancreatic islet cells in vitro dedifferentiation, due to prolonged culture timing and in specific conditions, has been demonstrated to lead to the loss of endocrine-pancreatic phenotype [1]. Indeed, in several previous studies, we and others have elucidated some of the mechanisms leading to the loss of endocrine-pancreatic phenotype and to the acquisition of an undifferentiated/mesenchymal-progenitor phenotype. Additionally, using in vitro β-cell lentiviral lineage tracing approach during human pancreatic islets in vitro dedifferentiation, it has been shown that β-cells can undergo to dedifferentiation process thus validating this model as a tool to study this phenomenon in β-cells [5,6]. Furthermore, some of the markers associated to the acquisition of β-cell undifferentiated/mesenchymal phenotype (e.g., Vimentin) have been recently demonstrated to be hyper-expressed in pancreatic islets analyzed from sections derived from pancreata of type 2 diabetic multiorgan donors [37]; these results further corroborated the validity of such in vitro dedifferentiation method. Therefore, using this in vitro model, we evaluated the expression profiles of microRNAs in human native islets and in dedifferentiated islet cells in order: (i) to gain further insights into the molecular mechanisms of dedifferentiation; (ii) to uncover potential biomarkers able to identify dedifferentiating/dedifferentiated islet cells from fully mature cells; (iii) to identify putative therapeutic targets in order to reverse the dedifferentiation process and to re-establish a fully functional β-cell.

By employing an unbiased profiling approach, we found that most of the differentially expressed microRNAs were downregulated (110/123, >80%) upon human pancreatic islet cells dedifferentiation, while only 13 were significantly upregulated. This is in line with microRNA expression variations usually observed during loss/acquisition of phenotype, where microRNAs buffer gene expression and restrain or facilitate cell fate decision [20]. Indeed, the set of identified downregulated microRNAs includes also those highly expressed in islets or in β-cells, which are reported to be responsible for the maintenance of endocrine-pancreatic phenotype by targeting “disallowed genes”, thus repressing abnormal cell responses or phenotypic changes [38,39]. This is the case for miR-30 and miR-200 families, previously reported to be involved in epithelial cells phenotype maintenance by repressing mesenchymal phenotype markers (e.g., miR-30 targets Vimentin and Snail) [24], or miR-375, miR-7a, and miR-9 which, more specifically, have been demonstrated to finely control β-cell function [40,41,42]. Overall, the set of 110 downregulated microRNAs during in vitro dedifferentiation (Supplementary Figure S3) may represent a microRNA core network needed by islet cells to maintain a mature phenotype and to specify their function.

From the other side, the 13 upregulated microRNAs may represent novel biomarkers of islet cells dedifferentiation and may target key genes whose repression facilitates the acquisition of an undifferentiated phenotype or involved in endocrine pancreatic cell functions. Particularly, the global analysis of their predicted target genes, highlighted a significant role in cell–cell adhesion or cell–cell signaling, whose function is essential for the maintenance of epithelial phenotype. Importantly, among the 13 upregulated microRNAs, we identified some of them previously addressed as pivotal for the acquisition and the modulation of mesenchymal phenotype during EMT process; these include: miR-99a, miR-100, miR-214, miR-137, miR-337-3p, miR-199a-3p/5p, and miR-708 [43,44,45,46,47,48]. Of note, some of them have also been previously associated to islets and/or β-cell function and dysfunction in diabetes; indeed, miR-199a has been demonstrated to be upregulated in pancreatic islets from ob/ob and db/db diabetic mice [42], in which β-cell dedifferentiation has been clearly established. MiR-708 has been recently reported to be upregulated in β-cells during metabolic/inflammatory stress conditions; moreover, it has been demonstrated to control the expression of neuronatin (Nnat) [49] whose expression was found reduced upon dedifferentiation in the present work. MiR-337 expression has been correlated to glucose-responsiveness of MIN6 β-cells [50], thus suggesting its potential role in the regulation of insulin secretion and β-cell function. Collectively, these data suggest that during islet cells dedifferentiation some upregulated microRNAs may induce β-cell dysfunction and EMT entry, thus rendering them strongly involved in the loss of β-cell phenotype.

Interestingly, among upregulated microRNAs we identified those belonging to the miR-302s family. MiR-302s are specifically expressed in pluripotent/undifferentiated stem cells, where they control several aspects of stemness [26]. Indeed, they have been reported to be controlled by stem-cell associated transcription factors Oct3/4 and Nanog, but also by transcription factors associated to Wnt/β-catenin signaling (Tcf/Lef) [28,31]. Additionally, overexpression of miR-302s microRNAs have been reported to be sufficient to reprogram somatic cells, thus inducing generation of induced-pluripotent stem cells (IPSCs) [51]. Here, we showed that miR-302s are upregulated during human pancreatic islet cells dedifferentiation and are associated to the loss of islets/β-cell phenotype. Using a bioinformatic approach followed by experimental evaluation of target genes expression, we demonstrated that upregulation of miR-302s was associated to the downregulation of target genes mostly involved in cell–cell adhesion and β-cell phenotype control (NeuroD1 and Kat2b). Additionally, we explored the potential cues which lead or contribute to the activation of miR-302 expression; specifically, we suggest that re-establishment of cell–cell contacts inhibits miR-302 expression. These evidence were corroborated by two different experimental settings, employing: (i) a forced cell–cell contact re-establishment by culture density experiments; (ii) the induction of a re-differentiation program which primarily led to the formation of pseudo-islets characterized by high cell–cell contacts. We hypothesized that disruption or re-establishment of cell–cell contact may modulate β-catenin signaling through the expression of E-cadherin, which, in turn, can bind β-catenin thus modulating the interaction with the downstream factors [34]. Therefore, the reduction of E-cadherin may enhance the availability of β-catenin to interact with downstream effectors, thus allowing the activation of miR-302s expression potentially mediated by Tcf/Lef factors. On the contrary, re-establishment of cell–cell contacts may reduce the bioavailability of β-catenin, thus reducing miR-302s transcriptional activation. The contribution of cell–cell contacts to miR-302s expression has also been demonstrated using a specific re-differentiation program; during such process we detected also the re-expression of one miR-302s target gene (NeuroD1), thus suggesting that miR-302s may target NeuroD1 and that the downregulation of such a microRNA cluster may remove the post-trancriptional inhibition and allow NeuroD1 upregulation [1].

Although establishment or disruption of cell–cell contacts have been reported to modulate Wnt signaling pathway, potentially leading to miR-302 transcriptional modulation, we cannot exclude that additional cues may activate others specific signaling which, in turn, can control miR-302 transcription. Additional experimental evidences, focusing on the direct modulation of Wnt signaling pathway using inhibitors or activators, are needed in order to verify the contribution of this pathway in the transcriptional activation of miR-302 cluster genes.

Further evidence of miR-302s involvement in β-cell fate control has been provided by microRNA profiling during human IPSCs differentiation toward endocrine-pancreatic phenotype; indeed, we demonstrated that miR-302s expression was reduced during endocrine pancreatic differentiation [20], thus suggesting that the backward process (dedifferentiation) is likely to enhance their expression.

Although our data, and other previous published evidence, pointed to an important role for miR-302 during dedifferentiation process and in the maintenance of undifferentiated phenotype, we cannot decipher whether miR-302s activation represent a direct consequence of dedifferentiation mechanism or a specific triggering factor of such process. Previous studies indicated miR-302s overexpression in mature cells, without any other additional factors, was able to induce the loss of mature phenotype and the acquisition of an undifferentiated/stem-cell like one; therefore, it is not unlikely that miR-302s upregulation alone may strongly contribute to the loss of β-cell phenotype. Therefore, additional studies are strongly needed in order to clarify whether miR-302s upregulation is a prerequisite for dedifferentiation triggering or is merely a consequence of the alteration of a more complex molecular network.

Another important point would be to assess whether those microRNAs found to be differentially expressed during in vitro dedifferentiation are mirrored by a similar differential expression in pancreatic islets derived from T2D donors and specifically selected based on their degree of dedifferentiation; this could be accomplished by laser capture microdissection and selection of those islets containing dedifferentiated cells vs. those retaining a fully mature phenotype.

The detailed uncovering of these molecular mechanisms and the potential confirmation of their involvement in diabetes may open the way for future therapeutic approaches involving the use of microRNA modulators; indeed, the use of inhibitors of miR-302s family members (or other microRNAs upregulated during dedifferentiation) may re-establish the correct molecular homeostasis, thus reducing the consequences of miR-302s hyperexpression and, potentially, restore β-cell function. Moreover, the low/null expression of miR-302 in mature native pancreatic islets represents a therapeutic advantage, permitting the desired inhibition only where miR-302 is hyperexpressed thus having no adverse off-target effects where miR-302 is not expressed (i.e., in not dedifferentiated cells).

In conclusion, these data demonstrate that microRNAs belonging to the miR-302 cluster are associated to islet-cell/β-cell dedifferentiation—i.e., a phenomenon involved in T1D and T2D development—suggesting that such group of microRNAs and their related target genes may represent novel candidate therapeutic targets in diabetes mellitus.

## 4. Materials and Methods

### 4.1. Human Pancreatic Islets Isolation and In Vitro Islet Dedifferentiation

Human pancreatic islets from non-diabetic multiorgan donors (see Supplementary Table S1) were isolated using pancreas collagenase enzymatic digestion and gradient separation as previously described [1]. Some pancreatic islet preparations were hand-picked and then used for RNA extraction (*Hi native*); others were hand-picked and then cultured to induce de-differentiation (*Dediff. Hi*) (see Supplementary Table S1). Dedifferentiation was induced as previously described [1]. Briefly, 50 hand-picked islet equivalents (IEQ), without dissociation, were cultured in 100 mm plastic tissue culture dishes (Falcon; Becton Dickinson, San Jose, CA, USA) in growth medium (modified RPMI 1640 medium (11.1 mM glucose) (Sigma Aldrich, St. Louis, MO, USA) supplemented with 10% FBS (Stem Cell Technologies Inc., Vancouver, BC, Canada), 2 mM l-glutamine, 100 U/mL penicillin, 100 mg/mL streptomycin, 250 ng/mL amphotericin B, (Sigma Aldrich) and maintained at 37 °C in 5% CO_2_ and 95% humidified air. After 15 days of culture, adherent dedifferentiated islets were detached with 0.25% trypsin-2 mM EDTA (Sigma Aldrich) and seeded at a density of 12,000 cells/cm^2^ for two passages and then subjected to downstream analysis. Dedifferentiated and expanded islet cells were re-differentiated as previously described [1] and analyzed at different time-points of differentiation (3 days, 7 days, 11 days, 15 days, 18 days, 21 days), or cultured at low density (4000/cm^2^) and high density (100,000/cm^2^) in standard growth medium in 6-well adhesion petri dishes for 48 h, then detached and analyzed for microRNA expression levels.

### 4.2. RNA Extraction and Quality Evaluation

Total RNA, including small RNA fraction, was extracted using miRNeasy Mini Kit (Qiagen, Valencia, CA, USA) and treated with DNase I using RNase-free DNase set (Qiagen) to eliminate genomic DNA. RNA quality was assessed using Agilent Bioanalyzer 2100 (Agilent Technologies- Santa Clara, CA, USA); RIN value ≥6 was considered acceptable for Taqman Array Microfluidic cards and stem–loop RT-qPCR analysis.

### 4.3. MicroRNA Expression Profiles Using Taqman Array Cards

MicroRNA expression profiling was performed using Taqman™ MicroRNA Array Human Panel A + Panel B v2.1 (Life Technologies, Carlsbad, CA, USA) in order to evaluate the expression of 768 microRNAs. Megaplex™ Reverse Transcriptase reaction, was performed according to the manufacturer’s protocols (Life Technologies) using 500 ng of total extracted RNA. ViiA7 PCR instrument platform was used to analyze Taqman array cards and Expression Suite 2.1 software (Life Technologies, Carlsbad, CA, USA) was used to evaluate amplification plot efficiencies and to analyze data. Analysis was performed by using 2^−∆Ct^ method following normalization with small nuclear RNAs, RNU44 and RNU48.

Hierarchical clustering analysis plot was computed in order to obtain a global view of microRNA expression levels and to identify clustered group of microRNAs. Differentially expressed microRNAs were identified by performing a Volcano-plot analysis by applying a cut-off fold change of >2.5 (upregulated) or <0.4 (downregulated) and a statistical cutoff of FDR corrected *p*-value of *p* < 0.05 using Student’s *t*-test on normalized ∆Ct values. Hierarchical clustering analysis plot and volcano plot were elaborated using Spotfire 5.0 (Tibco, Somerville, MA, USA) and GraphPad 5.0 (GraphPad Prism, La Jolla, CA, USA), respectively.

### 4.4. MicroRNAs Stem–Loop Single Assay RT-qPCR

In order to analyze microRNA expression in single assay reaction, 10 ng of extracted RNA from six samples of native human islets and from seven samples of islet-derived dedifferentiated cells were subjected to reverse transcription reaction, performed using stem–loop reverse transcriptase protocol followed by real-time PCR using specific Taqman™ microRNA expression assay in 96-well plate (all from Life Technologies), according to manufacturer’s suggestions. Ct values were analyzed by using Expression Suite 2.1 software and normalized using endogenous RNU44 and RNU48.

### 4.5. Predicted Target Genes Bioinformatic Analysis

In order to investigate the main functions in which differentially microRNAs were involved, we firstly performed a bioinformatic analysis on Targetscan 6.2 (http://www.targetscan.org/vert_61/). Among predicted target genes we selected 196 genes based on their function (differentiation, proliferation, cell adhesion). A further deep bioinformatic functional analysis was performed by using DAVID 6.7 algorithm (https://david-d.ncifcrf.gov/); based on gene ontology categories and *p*-values, a final selection of 92 predicted target genes that putatively play an essential role in human islets development and function were identified.

### 4.6. Genes Expression Analysis

Expression analysis of endocrine-pancreatic and undifferentiated/mesenchymal genes expression was performed using Improm-II reverse transcriptase reaction protocol (Promega, Madison, WI, USA), followed by Taqman Gene expression assays analysis using real-time PCR (Life Technologies). 250 ng of total RNA/reaction were reverse transcribed and 25 ng of corresponding cDNA were employed to analyze each selected gene.

Identified and predicted 92 target genes (retrieved using the bioinformatic approach described above) were analyzed using Taqman Array 96 well Fast plate, specifically designed in a custom format (Life Technologies), containing lyophilized corresponding Taqman Gene expression assays. In this case, 500 ng of total RNA from each sample was reverse transcribed using Improm-II reverse transcriptase reaction. Then, a master mix containing Universal PCR Master Mix II, RNAse-free water and 500 ng of total cDNA were added to each well and then analyzed following manufacturer’s protocol (Life Technologies). GAPDH and β-Actin housekeeping genes were adopted to normalize gene expression results.

### 4.7. Statistical Analysis

Statistical significance was determined using Mann–Whitney non-parametric U test or two-tailed paired *t*-test (GraphPad Prism 5.0). P values less than 0.05 (*p* < 0.05) were considered statistically significant.

### 4.8. Ethics Statement

Pancreata were collected after informed consent was obtained in writing from family members. The islet isolation center has permission to prepare isolated islets and to use them for scientific research if they are not suitable for clinical islet transplantation, in accordance with national laws and our institutional ethical rules (Comitato Etico per la Sperimentazione dell’Azienda Ospedaliera Universitaria di Pisa).

## Figures and Tables

**Table 1:** Gene ontology (GO) terms segregation of 196 predicted target genes of upregulated microRNAs. GO term category, GO ID and specification, number of genes (count), and *p*-value of each specific GO term is reported.

Category	Term	Count	*p* Value
GOTERM_BP_FAT	GO:0007155 cell adhesion	41	5.18 × 10^−26^
GOTERM_BP_FAT	GO:0051094 positive regulation of developmental process	21	9.12 × 10^−15^
GOTERM_BP_FAT	GO:0006928 cell motion	19	8.66 × 10^−9^
GOTERM_BP_FAT	GO:0007267 cell–cell signaling	16	2.88 × 10^−5^
GOTERM_BP_FAT	GO:0040008 regulation of growth	14	1.16 × 10^−6^
GOTERM_BP_FAT	GO:0016055 Wnt receptor signaling pathway	11	4.45 × 10^−8^
GOTERM_BP_FAT	GO:0008284 positive regulation of cell proliferation	10	0.00329
GOTERM_BP_FAT	GO:0051046 regulation of secretion	6	0.016312
GOTERM_BP_FAT	GO:0032925 regulation of activin receptor signaling pathway	4	4.40 × 10^−5^
GOTERM_BP_FAT	GO:0046324 regulation of glucose import	3	0.024159
GOTERM_BP_FAT	GO:0032868 response to insulin stimulus	8	8.33 × 10^−6^

## Supplementary Materials

The following are available online at http://www.mdpi.com/1422-0067/19/4/1170/s1.
