# Supplementary material for: MicroRNA Expression Analysis of In Vitro Dedifferentiated Human Pancreatic Islet Cells Reveals the Activation of the Pluripotency-Related MicroRNA Cluster miR-302s
Source: Int J Mol Sci. 2018 Apr 12;19(4):1170. doi: 10.3390/ijms19041170 (PMC5979342; doi:10.3390/ijms19041170)
Supplement: Supplementary file 1 [file ijms-19-01170-s001.pdf]

Supplementary

MicroRNA Expression Analysis of In Vitro Dedifferentiated Human Pancreatic Islet Cells Reveals the Activation of the Pluripotency-Related MicroRNA Cluster miR-302s

Guido Sebastiani [1], Giuseppina Emanuela Grieco [1], Noemi Brusco [1], Giuliana Ventriglia [1], Caterina Formichi [1], Lorella Marselli [2], Piero Marchetti [2] and Francesco Dotta [1,*]

[1] Diabetes Unit, Department of Medicine, Surgery and Neurosciences, University of Siena, Fondazione Umberto Di Mario ONLUS c/o Toscana Life Science, 53100 Siena, Italy; sebastianiguido@gmail.com (G.S.); giusy.grieco.90@gmail.com (G.E.G.); noemibrusco91@gmail.com (N.B.); giulianaventriglia@gmail.com (G.V.); catefo@libero.it (C.F.)

[2] Department of Clinical and Experimental Medicine, University of Pisa, 56126 Pisa, Italy; lorella.marselli@med.unipi.it (L.M.); piero.marchetti@med.unipi.it (P.M.)

* Correspondence: francesco.dotta@alice.it; Tel.: +39-0577-586269

Supplementary Table 1. Non diabetic multiorgan donors characteristics; gender (F/M), age (years) and BMI (Kg/m^2) are reported. For each sample the specific analysis performed are indicated (Research use). *Hi native*: human pancreatic native islet preparations used for evaluation of genes and microRNAs without further in vitro processing; *Dediff. Hi*: human pancreatic islet preparation used for derivation of in vitro dedifferentiated islet cells.

Samples	Gender	Age	BMI	Research use
Hi native-1	F	79	23.9	MicroRNAs profiling, MicroRNAs single assay qPCR, Target Genes
Hi native-2	M	36	26.3	MicroRNAs profiling, MicroRNAs single assay qPCR, Target Genes
Hi native-3	M	75	24.2	MicroRNAs profiling, MicroRNAs single assay qPCR, Target Genes
Hi native-4	F	74	23.4	MicroRNAs single assay qPCR, Target Genes expression profile
Hi native-5	M	54	23.1	MicroRNAs single assay qPCR, Target Genes expression profile
Hi native-6	M	50	27.4	MicroRNAs single assay qPCR, Target Genes expression profile
Hi dediff-A	M	39	23.6	MicroRNAs profiling, MicroRNAs single assay qPCR, Target Genes
Hi dediff-B	F	46	27.2	MicroRNAs profiling, MicroRNAs single assay qPCR, Target Genes
Hi dediff-C	M	79	25.6	MicroRNAs profiling, MicroRNAs single assay qPCR, Target Genes
Hi dediff-D	F	44	24.2	MicroRNAs single assay qPCR, Target Genes expression profile
Hi dediff-E	M	41	24.8	MicroRNAs single assay qPCR, Target Genes expression profile
Hi dediff-F	F	42	22.4	MicroRNAs single assay qPCR, Target Genes expression profile
Hi dediff-G	F	50	24.9	MicroRNAs single assay qPCR, Target Genes expression profile

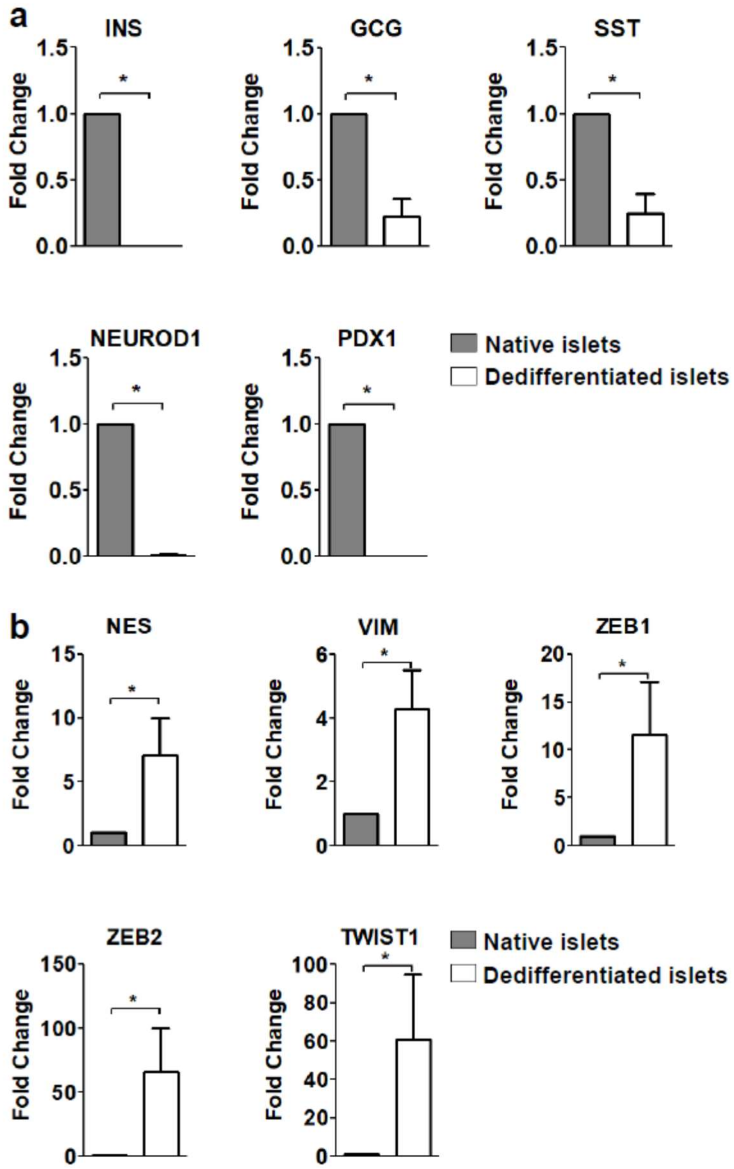

Supplementary Figure 1| Real Time PCR expression analysis of endocrine-pancreatic related genes (insulin, glucagon, somatostatin, Pdx1, Neurod1) (**a**) and of undifferentiated/mesenchymal phenotype associated markers (Nestin, Vimentin, Zeb1, Zeb2, Twist1) (**b**) in human islet-derived dedifferentiated cells (n=3) vs native human islets (n=3). Values are reported as fold change ± SD vs human native islet samples. p value $p<0.05$ student's t test.

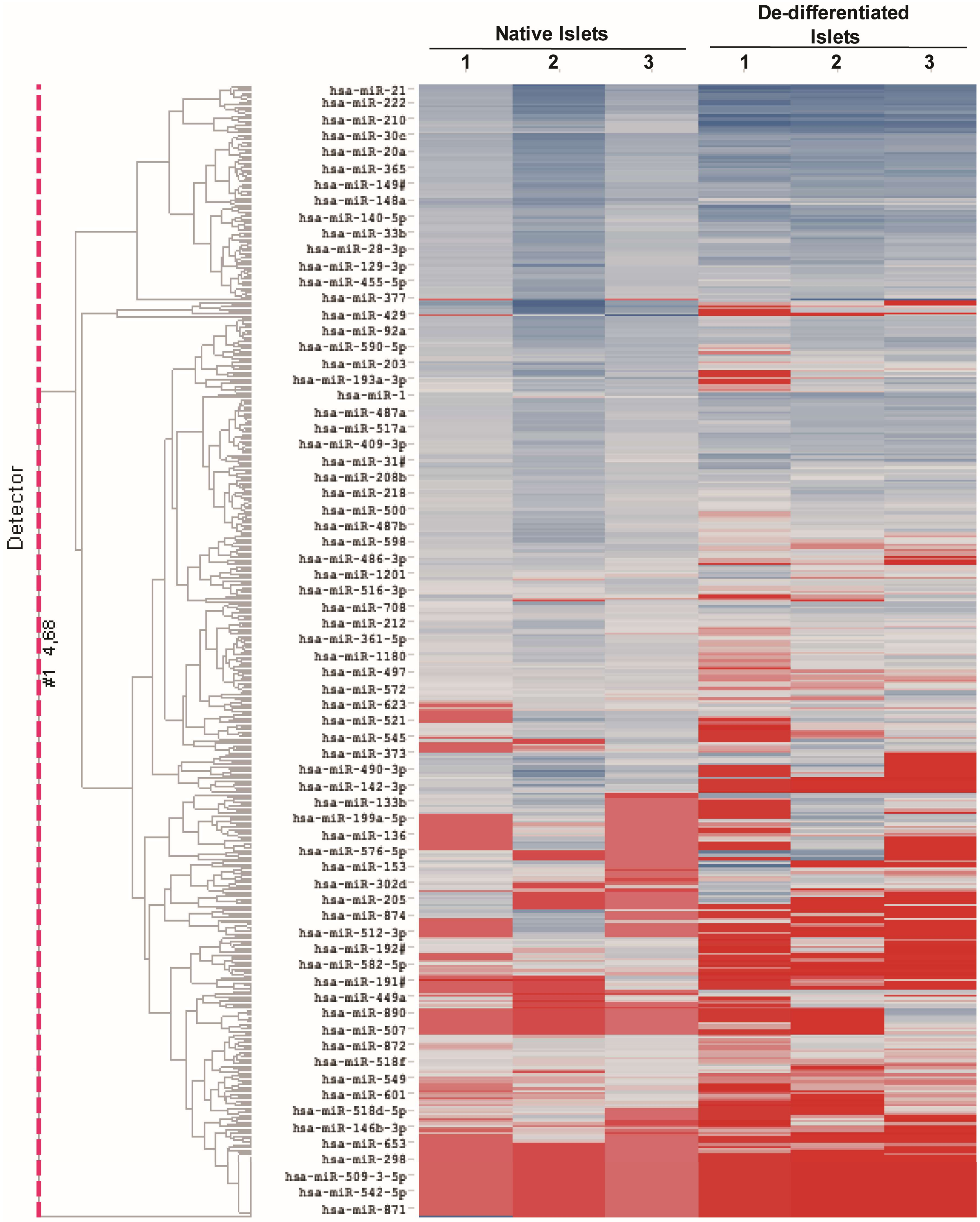

Supplementary Figure 2. Global Hierarchical clustering analysis of detected 342 microRNAs in human islet-derived dedifferentiated cells (n=3) vs native human islets (n=3). MicroRNAs expression level values are reported as normalized ΔCT and depicted in scale colours (from blue – highest expression- to red- lowest expression).

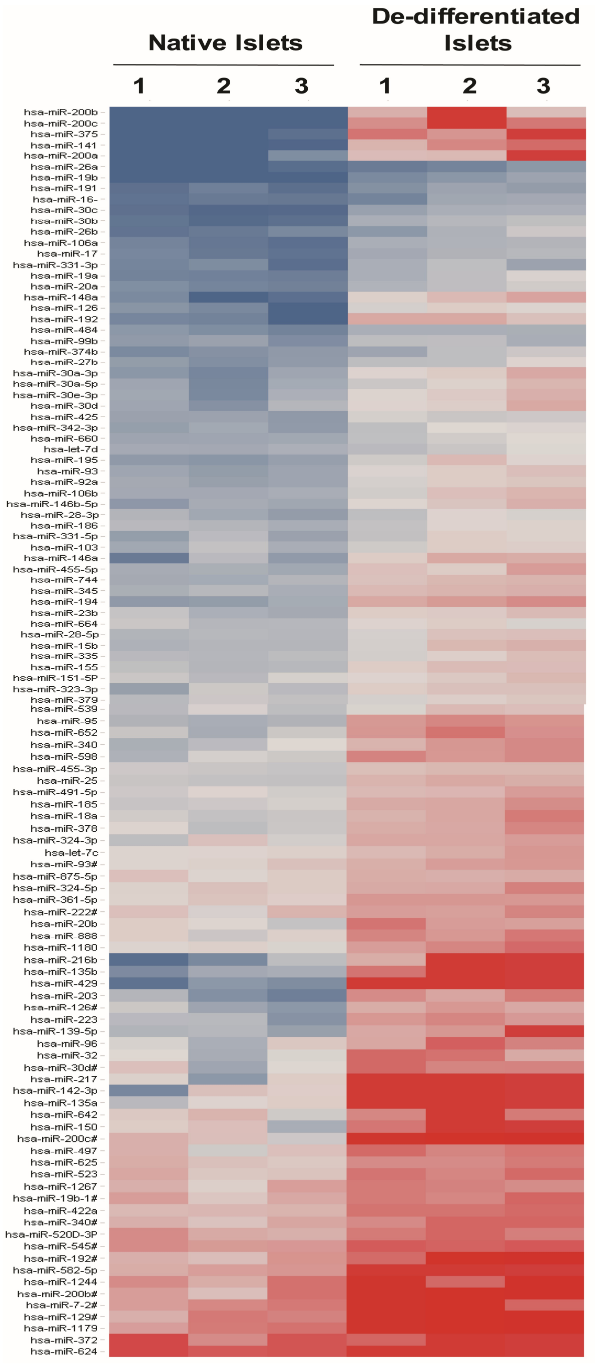

Supplementary Figure 3. Hierarchical Clustering Analysis of 110 downregulated microRNAs in human islet-derived dedifferentiated cells (n=3) vs native human islets (n=3)**.** The heatmap reports the expression levels of downregulated microRNAs upon dedifferentiation process of human native pancreatic islets. MicroRNAs expression level values are reported as normalized ΔCT and depicted in scale colours (from blue –highest expression- to red- lowest expression).

Supplementary Figure 4

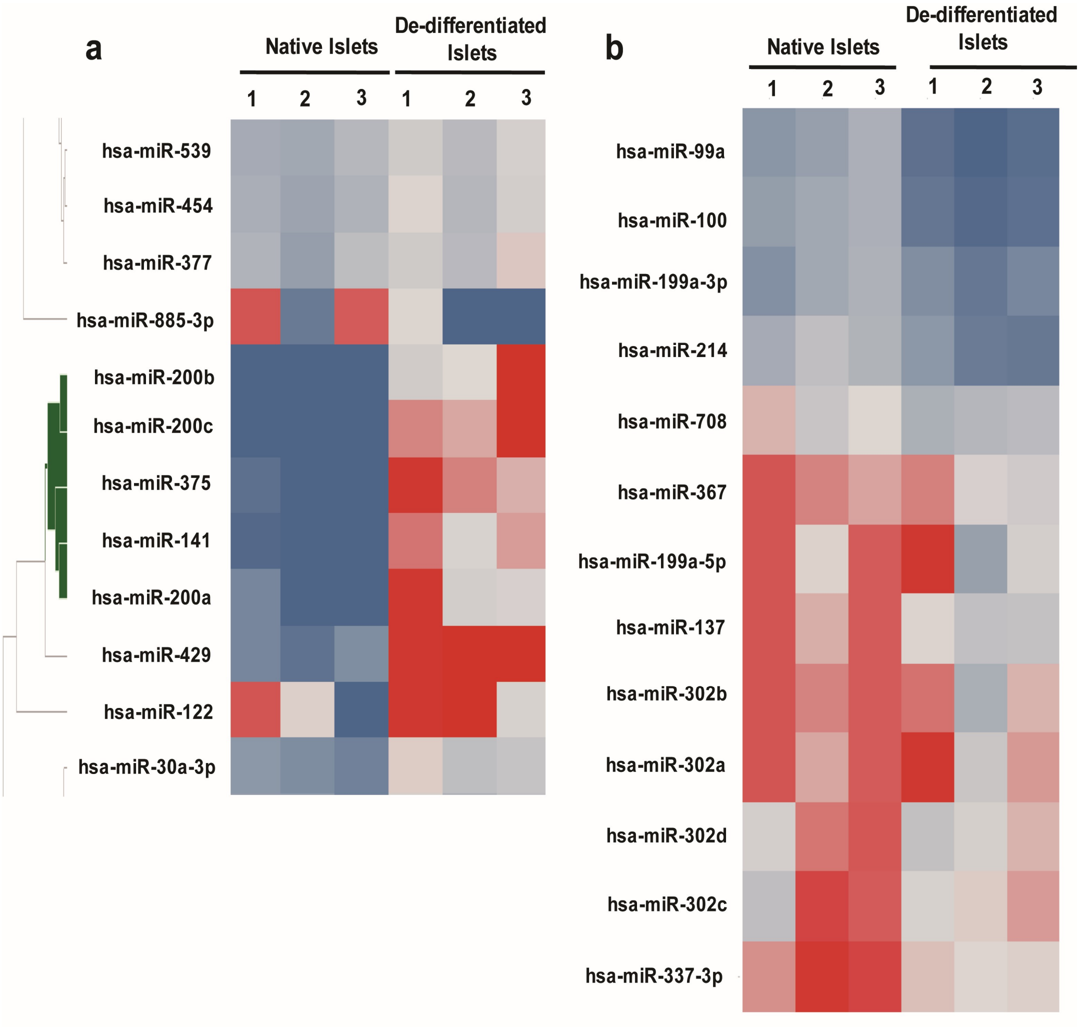

Supplementary Figure 4. (a) Detail of the global microRNA hierarchical clustering analysis showing miR-375 and miR-200 microRNAs (cluster highlighted in green)**. (b)** Detail of microRNA hierarchical clustering analysis reporting the upregulation of the 13 identified microRNAs. MicroRNAs expression values are reported as ΔCt values in scale colours (from blue –highest expression- to red- lowest expression).
